# Medicine Shortages: An Algorithm for Evaluating the Substitution with Equivalent or Alternative Products

**DOI:** 10.3390/healthcare13101139

**Published:** 2025-05-14

**Authors:** Gabriele Caviglioli, Giuliana Drava, Laura Pivetta, Carmine Di Meco, Eugenia Livoti, Gabriella Paoli, Sara Baldassari, Giorgia Ailuno, Maria Paola Franchina, Alessandro Bonsignore, Domenico Di Giorgio, Barbara Rebesco

**Affiliations:** 1 Department of Pharmacy, University of Genova, Viale Cembrano 4, 16148 Genova, Italy; giuliana.drava@unige.it (G.D.); sara.baldassari@unige.it (S.B.); giorgia.ailuno@unige.it (G.A.); 2 IRCCS Ospedale Policlinico San Martino, Largo Rosanna Benzi 10, 16132 Genova, Italy; alessandro.bonsignore@unige.it; 3 Specialization School in Hospital Pharmacy (SSFO), University of Genova, Viale Cembrano 4, 16148 Genova, Italy; laurapivetta@gaslini.org (L.P.); mariapaolafranchina@gaslini.org (M.P.F.); 4 Ligurian Regional Health Service (A.Li.Sa.), Piazza della Vittoria 15, 16121 Genova, Italy; c.dimeco@asl1.liguria.it (C.D.M.); eugenia.livoti@alisa.liguria.it (E.L.); gabriella.paoli@alisa.liguria.it (G.P.); barbara.rebesco@alisa.liguria.it (B.R.); 5 Section of Legal and Forensic Medicine, Department of Health Sciences, University of Genova, Via Pastore 1, 16132 Genova, Italy; 6 Italian Medicine Agency (AIFA), Via del Tritone 181, 00187 Roma, Italy; d.digiorgio@aifa.gov.it

**Keywords:** drug shortage, drug substitution algorithm, drug unavailability, drug shortage management, standard terms, drug market

## Abstract

**Background/Objectives**: Drug shortages are a serious issue affecting health systems worldwide, determined by multiple causes including supply issues, regulatory limitations, and market distortions. The possible repercussions on patients may impair therapeutic efficacy. Despite numerous actions being implemented by regulatory authorities, including market monitoring, export restrictions, and temporary regulation mitigations, few instruments have been made available to help health operators find marketed alternatives to unavailable products. The aim of this work was to create an algorithm to find equivalent or alternative medicinal products available in a certain pharmaceutical market. Algorithm development and validation were performed using the medicinal products marketed in Italy. **Methods**: First, a newly assembled code, describing the active pharmaceutical ingredient by its Anatomical Therapeutical Chemical (ATC) code, and its dosage form by the European Directorate for the Quality of Medicines & HealthCare (EDQM) Standard Terms, was attributed to each marketed medicinal product. Then, the algorithm was set up to identify its possible equivalents or alternatives by assigning a score quantifying differences in Defined Daily Dose (DDD) per presentation unit and in characterizing Standard Terms. **Results**: The algorithm was validated on a randomized sample of medicinal products, proving to be able to identify appropriate equivalents or alternatives; moreover, it was tested in real conditions by submitting a survey to health professionals, who found this product to be reliable and useful. **Conclusions**: The developed algorithm may be employed as a rational tool to help health operators find solutions to drug shortages. This work highlighted some limits of the current ATC attribution that should be addressed by the competent authorities.

## 1. Introduction

There is a lack of a standardized operative definition of drug shortage globally, as stated by the experts in the sector worldwide, who either define it from the supply side or from the user side. The World Health Organization (WHO) in 2016 announced two definitions: shortage occurs “when the supply of medicines, health products, and vaccines identified as essential by the health system is considered to be insufficient to meet public health and patient needs” [1]. While on the demand side, a shortage occurs “when demand exceeds supply at any point in the supply chain and may ultimately create a stock-out at the point of appropriate service delivery to the patient if the cause of the shortage cannot be resolved promptly relative to the clinical needs of the patient” [2]. This problem is affecting many countries worldwide, and is experienced in relation to all types of drugs, with sterile injectable formulations, essential medicines, and emergency medicines being more susceptible [2]. In particular, it has been outlined that the medicines presenting the highest risk of shortages are medicinal products (MPs) characterized by a low price and manufacturing complexity [3]. Drug shortages can occur due to many factors, including supply issues, demand issues, and regulatory issues. Supply issues consist of manufacturing and quality problems, unavailability of raw materials, logistic problems, and business decisions. In contrast, demand issues include just-in-time inventory, unexpected higher demand for a product, and demand fluctuations due, for instance, to seasonal necessities. Finally, regulatory issues may lead to delays in drug approval [4,5]. Examples of commercial issues causing a lack of medicines include medicine withdrawals due to the presence of noxious materials (i.e., valsartan [6]) or drug misuse (i.e., semaglutide employed for obesity treatment [7]). The supply problems of medicines and starting materials have become more acute with the relocation of chemical and pharmaceutical production to countries with less impactful labour costs and environmental, socio-economic, and pharmaceutical-specific regulations. Moreover, as evidenced in the 2023 US Pharmacopeia (USP) Annual Drug Shortages Report [3], the geographic concentration of pharmaceutical production, particularly in China and India, increases the vulnerability of the drug supply chain [8]. More than 50% of global active pharmaceutical ingredient (API) production is concentrated in five producer countries, and this strong concentration makes the European supply chains extremely vulnerable and affected by security-relevant weaknesses.

Another problem of medicine shortage is linked to the mechanism for fixing the price of generic medicines, in which insufficient revenues can be determined, leading to the potential discontinuation of the marketing of the product by the interested company. In the European Union (EU), parallel trade has been identified as an additional risk factor for medicine shortages in low-price Member States (like Poland, Slovakia, Greece, and Spain), even if the number of studies regarding the correlation between parallel trade and medicine shortage is currently insufficient [9,10,11].

Moreover, new economic plans in pharmaceutical companies may also be responsible for limitations in drug supplies (i.e., low investments in low-profit drugs such as generics).

Patients are the stakeholders mainly affected by the consequences of shortfalls in medicine supply: besides suboptimal treatments, they may experience delayed care, extended hospitalization, surgery cancellations, etc. [2,12].

Moreover, drug shortages have a conspicuous economic impact. For example, the cost of the annual management of drug shortages in the United States might be approx. USD 416 million, to which a further USD 215 million is to be added for the purchase of alternative medications [13].

The management of drug shortages may include the following: restrictions of the use of current stocks, accelerated drug approval, use of medicines with minor defects that cannot normally be employed, and the extension of expiry dates. Several States have developed medical platforms, providing information to physicians, pharmacists, and final users about forthcoming shortages and their management, and guidelines to be applied at national and possibly at international levels, as frequently the responses from single countries, and even at the level of health facilities within the same country, are uncoordinated [2,13].

The main regulatory agencies, like the Food and Drug Administration (FDA), which has been researching this issue since 1999, and, more recently, the European Medicines Agency (EMA), have been studying the phenomenon to identify drug shortages and potential remedies, both adopting several measures to mitigate them [1,2].

EU regulations require the marketing authorization (MA) holder to notify the national agency of any temporary or permanent discontinuation of an MP’s marketing within the national territory no less than two months before the interruption, except in the case of unforeseeable and exceptional circumstances [14,15].

Countermeasures such as an appropriate management of communication on drug shortages and the promotion of the use of equivalent, imported, and compounded medicines are essential to convey accurate information, counter hoarding practices, and prevent supply tensions for medicines [16]. Besides these initiatives, a rational tool aiding healthcare professionals in the management of drug shortages might be highly beneficial.

A “Pilot Project on Drug Shortages in Regione Liguria”, involving various actors, including the Italian Medicines Agency (AIFA), the regional offices for drug policies of Regione Liguria (A.Li.Sa.), experts and trainees of the Specialization School in Hospital Pharmacy from the University of Genoa, and main professional stakeholders, was established with various objectives, including to provide informatic support to healthcare professionals in selecting the most suitable alternative when an MP is unavailable on the market. The development of an algorithm which allows for a comparison of the different MPs by using a novel code able to describe them might be helpful in pointing out available pharmaceutical alternatives to physicians.

The aim of this paper is to present the algorithm developed for this goal, which can provide a ranking of possible substitutes for a drug in shortage according to the availability of equivalent MPs, or alternative MPs with different dosages, dose unit numbers, pharmaceutical forms, or administration routes. This algorithm can fill a gap in day-to-day drug substitution decisions, being easily adapted to any market areas or healthcare systems. In the literature, a number of publications have dealt with drug shortages by analyzing causes, trends, and impacts in different national systems [17,18,19,20,21,22,23], but, to the best of our knowledge, no algorithms similar to ours have ever been disclosed, nor have similar rational tools been developed and implemented, and this underlines the novelty of this approach.

## 2. Materials and Methods

### 2.1. Data Source

Information on authorized MPs on the Italian market were sourced from the database Farmadati Italia^®^ (Piacenza, Italy) on 18 June 2024; this is an Italian database that provides information on products related to the pharmaceutical and healthcare sector which are authorized and marketed in Italy, including medicinal products both for human and veterinary use. The database is updated daily and currently contains approximately 30,000 MPs, about 2000 of which are over-the-counter (OTC). This database, also used by AIFA only for MPs for human use, is proprietary and access requires a subscription; no patient information is listed.

The MPs in shortage were sourced from the AIFA list [24] at the same date of 18 June 2024. On a regular basis of about twice a week, AIFA updates the complete list of MPs not or no longer available on the market, due to production or regulatory issues, discontinued marketing, or suspension. The list contains the following information: trade name of the medicine in shortage, active ingredient, pharmaceutical form, packaging, and name of the MA holder; start date and estimated end date of the shortage; reasons for the shortage; and suggestions and/or measures adopted by AIFA to mitigate the shortage. These data are non-sensitive, public, and no permission to use them is needed; no patient information is listed.

### 2.2. MP Code Description

All the MPs included in the database were multimodally classified using internationally recognized nomenclature: Anatomic Therapeutic Chemical (ATC) Classification, Defined Daily Dose (DDD), and European Directorate for the Quality of Medicines & HealthCare (EDQM) Standard Terms (ST) [25]. This newly assembled code, defined as above, was attributed to every MP Italian marketing authorization (AIC) number.

Five specific STs were used: Basic Dose Form (BDF, used to group together related pharmaceutical dose forms); Administration Method (AME, pharmaceutical dose form for administration to the patient after any necessary transformation of the manufactured dose form has been carried out); Intended Site (ISI, general body site at which a pharmaceutical product is intended to be administered); Release Characteristic (RCA, description of the timing by which an active ingredient is made available in the body after administration of the pharmaceutical product, in comparison to conventional, direct release of the API); and Transformation (TRN, procedure that is carried out in order to convert a manufactured item that requires such a procedure into a pharmaceutical product, i.e., from its manufactured dose form to its administrable dose form) [26].

### 2.3. Algorithm Development and Its Implementation

The different parameters used to describe each MP were combined into an equation, where different weights were assigned to each ST. This equation allowed for browsing the possible MPs and providing a list of alternatives, assigning them a score based on the similarity to the MP in shortage.

Microsoft Excel was selected as the primary tool for designing and developing an interactive dashboard due to its ability to integrate data management, its feasibility for the algorithm implementation, and the possibility to both visualize and export the obtained results. This choice proved advantageous, as Excel facilitates the handling of all the datasets analyzed, mainly provided as .xlsx files, also including the .ODS file provided by AIFA concerning drug shortages. This enabled an all-in-one solution for the manipulation and analysis of the data provided.

Leveraging Excel functionalities like Power Query, Power Pivot, and Visual Basic for Applications, data manipulation and recurrent updates were automated. Furthermore, Microsoft Excel allows to graphically represent the results and export them in PDF format, which ensures the intuitive but complete dissemination of analytical outputs.

These features made it possible to develop an interactive dashboard easily accessible by pharmacists and medical doctors, who could readily evaluate the algorithm effectiveness given a selected input of their choice and export the results for further analysis in the future.

### 2.4. Algorithm Validation

For the “internal validation”, a test set of items corresponding approx. to 3% (*n* = 598) of all items included in the Farmadati database (*n* = 20,884, only considering MPs for human use marketed in Italy) was randomly selected, where each ATC class (1st level) was represented according to the consumption percentage in Italy (Table S1) [27].

The algorithm was run on each item of the test set, returning several equivalents, if present, and alternative items. The list of items proposed was inspected for the correctness and completeness of the choice of equivalent MPs and for the appropriateness of the ranking of alternative MPs. Moreover, the responses were evaluated by several indicators measuring the similarity between each alternative and the reference item in terms of score, BDF and number of different STs; these 10 indicators (percentage of alternatives with DS score = 100%, in the range 90–99%, in the range 80–89%, and <80%; percentage of alternatives with the same BDF of the reference item; percentage of reference items with alternatives that differed by a maximum of 1 to 5 STs) were computed and analyzed by univariate statistical methods and by Principal Component Analysis (PCA) as multivariate exploratory data analysis.

For “external validation”, the algorithm, implemented by an Excel Microsoft macro, was tested in real conditions by a panel composed of 15 general practitioners and 3 hospital pharmacists, who received the alternatives proposed by the algorithm for newly listed AIFA shortage drugs on a weekly basis. At the end of the validation phase, each participant was asked to provide feedback by filling in an electronic form, judging the performance and the effectiveness of the algorithm.

## 3. Results and Discussion

The algorithm proposed here is designed based on the descriptive strings of the MPs, assembling internationally standardized codes for the identification of the API and the description of the characteristic pharmaceutical properties of the MPs, which, to the best of our knowledge, have never been used before in this context as is conceived here. The developed algorithm is applied and validated using MPs authorized for the Italian market and, in particular, provided by the Italian Health Service (Servizio Sanitario Nazionale, SSN). It is noteworthy that we use a localized Italian dataset as the only accessible source of MPs on the market available to us. The datasets of other countries may only change in terms of the quantitative composition (number of MPs), but in worldwide national administrative datasets the registration number of each marketed product is associated with the information useful for the application of the universally recognized codes ATC/DDD and ST. The combined code that we propose allows for the use of this or other future algorithms, enabling the interoperability among the different MP databases of different national health systems.

The algorithm provides a flexible tool to help operators identify potential substitutions in drug shortages, though additional region-specific adaptations or validations might be required, and can be managed by a computerized system. To consider the real value of the dataset used, it has to be considered that as of December 31, 2022, the population in Italy was 58,997,201 residents, of which more than 60% had received at least one prescription for drugs in the previous 12 months [28]. In the same year, public pharmaceutical expenditure represented 68.9% of total pharmaceutical expenditure with a value of EUR 23.5 billion.

The concept of the therapeutic equivalence of a drug has consolidated over the years at a global level, certainly thanks to the work carried out by the International Council for Harmonisation of Technical Requirements for Pharmaceuticals for Human Use (ICH), WHO, FDA, and EMA. The approach to the problem by the FDA was pioneering, with the “*Approved Drug Products With Therapeutic Equivalence Evaluations*”, now commonly known as the *Orange Book*, being published since October 1980 and currently on its 45th edition [29].

The equivalence-related terms and definitions used in this paper are those reported in the introduction of the *Orange Book*. Pharmaceutical equivalents are drug products in identical dosage forms and route(s) of administration that contain the same amount of the same API; pharmaceutical alternatives are drug products that contain the identical therapeutic moiety, or its precursor, but not necessarily in the same quantity or dosage form, or the same derivative; approved MPs are considered to be therapeutic equivalents if they are pharmaceutical equivalents for which bioequivalence has been demonstrated, and they can be expected to have the same clinical effect and safety profile when administered to patients under the conditions specified in the label. The concept of therapeutic equivalence applies only to MPs containing the same API(s) and does not encompass a comparison of different therapeutic agents used for the same condition: in this paper, this last case is indicated as the therapeutic alternative MP.

The developed algorithm, given an unavailable MP, allows for the pharmaceutical equivalents present on the market to be found and listed by attributing to them a degree of substitutability (DS) of 100%, together with the found pharmaceutical alternatives, sorted in decreasing order of DS.

The algorithm works by identifying the MP through the code conceived by the Authors as a union of the ATC code [30] with five of the STs proposed and managed by EDQM ver. 1.2.0-28 January 2019 [26], as listed in Table 1. The identification code is completed with the number of DDDs for the presentation unit (NDXUP), calculated as the number of DDDs referring to the single unit pharmaceutical dosage form (e.g., tablet, capsule) or referring to volume (mL) or weight (g) for liquids and solids in single and multiple dose forms (e.g., syrup, solution).

The EDQM ST code has been recognized as the leading system in pharmaceutical product description, initially drawn from the European Pharmacopoeia Commission for use in drug labelling, summary of product characteristics, and digital communication, as a result of the implementation of ISO 11239:2012 and ISO/TS 20440:2016 [31]. Since 2017, the scope of the ST database has widened to allow for the inclusion of different aspects, like adverse event reporting and clinical trials. It can be used for many other purposes in digital communication or pharmaceutical data analysis, or when an accurate description of an MP pharmaceutical characteristics is necessary. The algorithm presented here uses the five main or traditional STs: basic or generalized dosage form or group of related pharmaceutical dosage forms (BDF); Administration Method (AME); Intended Site or the site at which a pharmaceutical product is intended to be administered (ISI); Release Characteristic (RCA); and Transformation or procedure that must be carried out to convert a manufactured dosage form to its administrable dosage form (TRN). Each ST is associated with a four-digit numeric code. Table 2 shows an example of ST codification for some MPs containing risperidone, an atypical antipsychotic mainly used in schizophrenia and bipolar disorder.

Each code, like those reported in Table 2, is preceded by the five levels of the ATC code (for risperidone, ATC = N05AX08). For example, for the first item in Table 2, the code is reported in Table 3.

When the algorithm is queried with the MA number of a lacking or unavailable MP, as a first step, it converts the characteristics of the pharmaceutical product in the above-described code, and uses it to search in the database for the pharmaceutical equivalents (with the same digital string) and the alternatives (same ATC, but with some differences in the ST or NDXUP part of the digital string), returning a list of MPs in descending DS order.

The DS score for pharmaceutical equivalence is set at 100, a value from which penalties are deducted in the case of alternatives with differences from the factor classes of the compared MPs, as calculated in Equation (1):

DS = 100 − (penalty score _NDXUP_ + penalty score _STs_) (1)

For the class NDXUP, the maximum deduction is set at 10 points. For the ST classes, the maximum deduction is set at 80 points.

In Table 4, the criteria to attribute the penalty scores for any difference in the number of DDDs for the presentation unit (NDXUP) between the unavailable or lacking MP (lak) and its potential substitute MP (sub) are reported.

In identifying a pharmaceutical alternative, beyond some choices of score attribution that can be considered reasonable, even if arbitrary, it is preferred to give less weight to the difference in the dose contained in the pharmaceutical form compared to the other characteristics of the pharmaceutical form described by the STs. In fact, the maximum penalty of 10 is attributed to dosages of API which are very different from one of the lacking MPs (i.e., to dosages more than twice higher or less than twice lower).

For the ST class score, the lists in each ST are grouped together depending on the similarity of the characteristics or properties, attributing them a numerical value for each relative position (RP), as shown in Table 1. The distance between the RPs varies from zero, in the case of exact correspondence of the MPlak and MPsub STs, to a maximum value that is a function of the ST scale, which is the whole range of the RPs for that specific ST. Every relative difference in absolute value is normalized to 100. For example, for BDF, the normalized relative distance (NRD) between tablet (lacking) and syrup (alternative) was calculated as in Equation (2):

(2) $${\mathrm{NRD}}_{\mathrm{tablet}-\mathrm{syrup}}=\frac{|RP\;tablet-RP\;syrup|}{|maximum\;difference\;in\;relevant\;RP\;scale|}\times 100=41.30$$

Each contribution for ST is weighed with a different weight, as shown in Table 5.

The difference in weight factors among the STs is necessary to offset the strong leverage effect of STs that contain fewer terms, such as RCA and TRN, and at the same time to attribute to BDF, AME and ISI a minimum advantage in the selection criteria for the choice of pharmaceutical alternative. The calculated weighed contribution for the previous example is 41.30 × 0.46 = 18.998. To calculate the score of the ST class, the weighed NRD contribution of each of the five STs is normalized to 80, the maximum score reserved for this class, as in Equation (3):

(3) $$Total\;penalty\;score\;for\;ST\;class=\sum_{n}^{1-5}\frac{{NRD}_{n}\times {wf}_{n}}{100}\times 80$$

During the development and evaluation of the algorithm, the problem of the ambiguous codification of the ATC of combination products, or otherwise defined fixed combinations (FCs), was afforded. In the Guidelines for ATC classification and DDD assignment 2024 [30], the FCs containing two or more APIs belonging to the same fourth level are normally classified using the fifth level codes 20 or 30; the FCs containing two or more APIs not belonging to the same fourth level are normally classified using the 50-series as the fifth level; and FC products containing psycholeptic drugs not classified as N05 or N06 are classified at separate fifth levels using the 70-series. It may be difficult to establish a rule for all FCs and it is not easy to decide how an FC should be classified. For example, an MP containing an analgesic and a tranquillizer used primarily to ease pain should be classified for its main therapeutic indication, i.e., as an analgesic; likewise, an FC of an analgesic and an antispasmodic drug will be classified in A03 (drug for functional gastrointestinal disorders). This algorithm, in order to run correctly, needs an unambiguous recognition of the APIs in FCs. Therefore, only as a proof of concept of the operation of the algorithm, for FCs with ambiguous codes, fictitious ATC codes are used, reporting as fourth level the one of the API with the main therapeutical effect and using for the fifth level a number in the range from 99 to 80 that has never been used before. Another criterion for the fourth level could also be to refer to the component present in a larger quantity, but, in this case, there is the possibility of losing therapeutic information. Some examples of this new attribution are reported in Table 6, along with the original ATCs, to identify the FCs unambiguously. For example, in the case of a combination birth control pill (desogestrel and ethinylestradiol), the code G03AA09 is unambiguous, while for A03DB04, the ATC/WHO classification describes butylscopolamine and analgesics without indicating the analgesic drug, so for the FC of butylscopolamine and paracetamol the fictitious code A03DB95 is chosen.

In the case where some of these codes have already been used to describe an API molecule, for the fifth level, a letter of the English alphabet (26 characters) associated with a number chosen in the range from 0 to 9 could be used, thus providing 260 unique codes for FCs with the same ATC; by exchanging the position of the letter with the number, the possibility of univocal identification could double to 520. This notation at the fifth ATC level would also allow for the recognition of fixed combinations, because in the ATC code the fifth level is represented only by numbers. Anyway, this aspect should be addressed at the international level by the WHO Collaborating Centre for Drug Statistics Methodology [32], with the considerable advantage of having an unambiguous code for each type of MP.

Another issue that arises is due to there being no definition of DDDs for FCs. To overcome this issue, the DDDs for FCs are calculated as the sum of the DDDs of the APIs (referring to the solid dosage unit, or volume or weight) in the FC. This allows for a specific DDD to be assigned to the FC, which enables the assignment of different scores, using Equation (4):

NDXUP_FC_ = Dosage_API1_/DDD_API1_ + Dosage_API2_/DDD_API2_ (4)

The probability to attribute the same NDXUP to two APIs in an FC is remote, because two FC MPs marketed with inverted dosage would need to exist, which seems to be unrealistic.

In Table 7, the NDXUP sum of the DDDs of the two APIs in the FCs is reported.

Also, for electrolytic solutions, the same issue has arisen, so the use of a univocal fictitious ATC code that unambiguously identifies the unique qualitative–quantitative composition of the MP and attributes 1 as a formal value to the sum of NDXUP is conceived (Table S2).

In Table 8, as an example, the output of the Excel macro implementing the algorithm searching for a film tablet containing 2 mg of risperidone (Italian MA number 037599230), being lacking/unavailable on the Italian market is reported.

The Excel macro result returns 32 items as being potential MP substitutes. The first four are equivalent pharmaceutical products, having a DS score of 100. From item 5 to item 19 in ranking order, the macro finds alternative MPs differing only for dosage, but with identical STs: in this case, the score attributed by the algorithm favours (98%) MPs with half the required dosage (double intake), with respect to those with twice the content (96%; symmetrical division of the tablet) and those containing 3 mg (92%; only two-thirds of the entire tablet must be taken). In any case, the algorithm considers an MP with a DS score > 90% as a potential candidate for substitution. Scrolling the list of the outputs, it can be observed that the DS score decreases from oral tablets to oral solutions (still > 80%), and decreases even more to parenteral solutions (<60%).

The validation phase includes two different stages: an “internal validation”, conducted using the algorithm internal database, and an “external validation”, involving the recruitment of a “panel group” to evaluate the algorithm responses from a clinical perspective.

Based on the analysis of the outcomes of the internal validation phase, the algorithm provides reliable responses for all types of MPs. Only 13 of the 598 items used as the validation test set had no alternative, corresponding to 2%.

**Figure 1:**
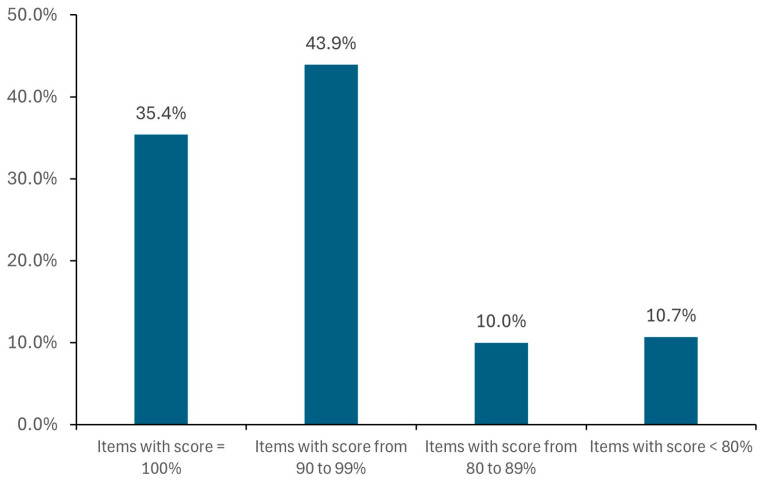
Results of validation performed on a test set of approx. 600 MPs belonging to different ATC classes, including 345 different APIs or FCs: 79.3% of the alternatives found had a DS score ≥ 90.

For each item, a different number of alternatives are found (from 1 to more than 100, e.g., for ibuprofen, pantoprazole, and paracetamol), in most cases with DS score ≥ 90% (Figure 1).

The number of items examined during the test involving the randomized sample of 598 MPs authorized in Italy is noteworthy. Considering that the macro suggests a variable number of alternative items for each searched MP (from a few items to several tens of items), there is a different multiplication factor for each of the 598 searched MPs; thus, the evaluation is performed on a number of alternative items largely higher than 598.

**Figure 2:**
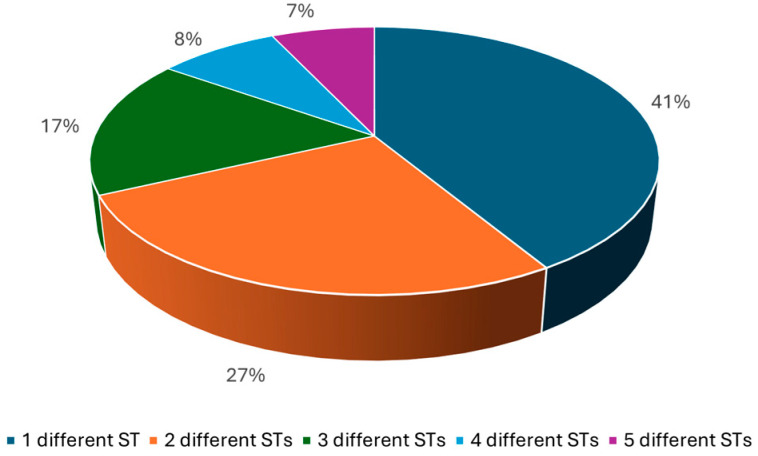
Results of the validation performed on a test set of approx. 600 MPs belonging to different ATC classes, including 345 different APIs or FCs: percentage of reference items with alternatives that differ by a maximum of one to five STs.

Most of the alternatives (80%) have the same BDF as the reference item; only 15% of the reference items have alternatives differing for four or five STs (Figure 2).

**Figure 3:**
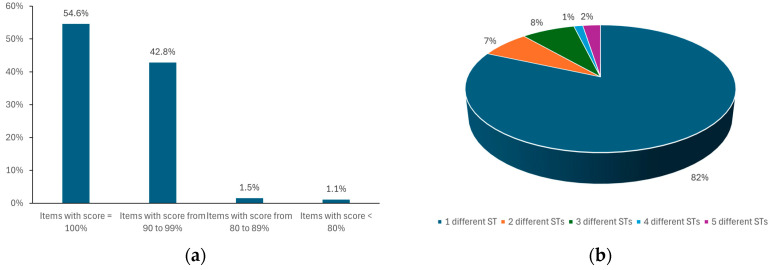
Results of validation performed on a test set of 161 MPs belonging to the cardiovascular system (ATC first level = C), including 79 different APIs or FCs: (**a**) 97.4% of the alternatives found have a score ≥ 90%; (**b**) percentage of reference items with alternatives that differ by a maximum of one to five STs.

The analysis, performed separately on each ATC class, highlights the best performance for the ATC class of the cardiovascular system, which is the class with the highest % of drug consumption in Italy (Figure 3).

**Figure 4:**
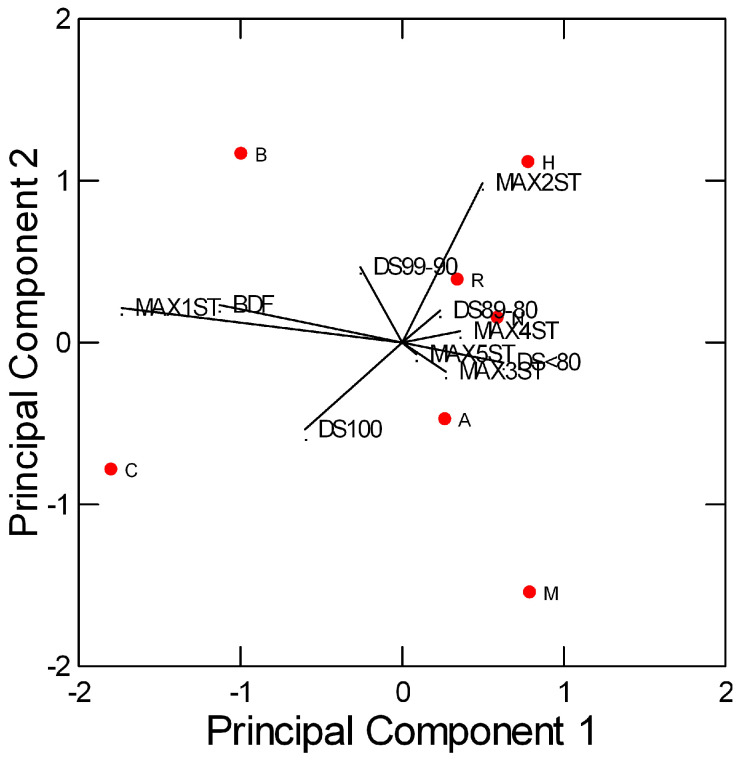
Results of PCA performed on the ten performance indicators of the seven ATC classes (C, A, N, R, M, B, H) at the highest % of drug consumption. The performance indicators considered are MAX1ST, MAX2ST, MAX3ST, MAX4ST, MAX5ST (percentage of reference items with alternatives that differ by a maximum of one to five STs, respectively); BDF (percentage of alternatives with the same BDF of the reference item); DS100, DS99–90, DS89–80, DS < 80 (percentage of alternatives with DS score = 100%, in the range 90–99%, in the range 80–89%, and <80%, respectively).

In order to visualize, in one plot, the results of the internal validation, Principal Component Analysis (PCA) is performed, simultaneously considering the ten performance indicators and showing the ATC classes that behave similarly (Figure 4). The analysis is limited to the seven ATC classes at the highest % of drug consumption (Table S1) to evaluate the algorithm’s performance on an acceptable number of MPs.

The first two Principal Components explain 78.6% of the total variance in the data. The C (cardiovascular system), A (alimentary tract and metabolism), B (blood and blood-forming organs), and R (respiratory system) classes show the highest number of alternatives, with DS scores > 90%. For B and C, respectively, 99% and 98% of the alternative items have the same BDF of the reference drug; moreover, most of the reference items have alternatives that differ by a maximum of one ST (60% and 82%, respectively). The algorithm seems to underperform for class A, where 23 and 29% of the reference items have alternatives that differ by three STs and by five STs, respectively. This can be attributed to several drugs in the test set (e.g., pantoprazole, omeprazole) for which the list of alternatives includes both oral and parenteral dosage forms.

However, besides the demonstrated efficacy of this algorithm, the final substitution choice requires an in-depth clinical evaluation: it is up to the physician to decide whether a proposed alternative is clinically feasible, especially in the case where the missing and the proposed product present considerable differences in terms of dosage or formulation composition. We acknowledge that this algorithm can help with, but not replace, a healthcare operator’s professional experience. Therefore, an external validation is mandatory.

For external validation, the feedback document containing short-answer questions (YES/NO/OTHER) received from a panel of 18 professionals is shown in Table 9, together with the evaluation results. A broader testing plan, including a higher number of healthcare practitioners belonging to a multi-regional area, is being outlined to confirm the significance of the results obtained from the preliminary external validation.

## 4. Conclusions

The developed algorithm tries to meet the needs of being a reliable, rational instrument to help health systems find adequate replacements for currently unavailable MPs. This tool can be considered a valuable support in the decision-making process of healthcare professionals.

The innovative aspect of this work lies in the application of the WHO ATC codes, of the DDD codes, and of the EDQM Standard Terms to codify MP identifiers usable by IT systems and to describe the APIs, the pharmaceutical characteristics of the dosage form, and the drug strength.

During validation, the algorithm proved to be able to find all the pharmaceutical equivalents of the indicated MP.

Some of the critical points found during algorithm development, such as the univocal description of FC products or electrolyte solutions and the comparison of dose strength between solid and liquid forms and mono- or multidose presentation, are tentatively addressed, though a standardization is needed at an international level.

The algorithm is structured to be flexible by being able to change either the values of arbitrary criteria or by choosing different weight factors or attributing different penalty scores. This feature allows for further adjustments to improve the algorithm performance. Studies to evaluate the robustness of the algorithm, considering sensitivity analyses or alternative scoring scenarios, are being planned.

In spite of using Italy-focused data, the use of universally recognized codes such as ATC/DDD and ST in the algorithm enables interoperability among different MP databases of different national health systems, provided that they include sufficiently detailed ATC/DDD and dosage form descriptions.

The preliminary validation, although limited in sample size, vouches for the algorithm clinical utility. An extension of the number of testing practitioners, even involving those belonging to different regulatory areas, is being planned.

The implementation of this algorithm in practical use requires overcoming some challenges, like its transfer to an informatic platform which is easily interfaceable or integrable with the most common IT health systems, its acceptance by clinicians and pharmacists, and its compliance with regulatory constraints.

This algorithm could also be used in different fields, for example, in Pharmacovigilance, Pharmacoutilization, Pharmacoepidemiology, and Pharmacoeconomics. Moreover, it can be employed in preventive risk analysis to highlight potential critical issues in an MP database of a national health system, highlighting the items that have no or few valid substitutes.

For large-scale implementation or iterative improvements, the collaboration with international standard-setting bodies, like WHO and EDQM, and the involvement of more stakeholders, will be necessary.

## Figures and Tables

**Table 1:** Standard Terms (STs) by EDQM: BDF, Basic Dose Form; AME, Administration Method; ISI, Intended Site; RCA, Release Characteristics after administration; TRN, Transformation to administrable dose form. Each ST is identified by an ID number (ST-ID) and ordered according to the proximity of the described properties by assigning it a numerical value corresponding to its relative position (RP).

BDF			AME			ISI			RCA			TRN		
ST-ID		RP	ST-ID		RP	ST-ID		RP	ST-ID		RP	ST-ID		RP
0069	Tablet	1	0019	Swallowing	1	0031	Oral	1	0047	Conventional	1	0042	No transformation	1
0058	Lozenge	2	0018	Sucking	2	0032	Oromucosal	2	0045	Prolonged	3	0038	Dilution	3
0051	Capsule	3	0014	Orodispersion	3	0023	Dental	3	0046	Modified	6	0040	Dissolution	5
0050	Cachet	3.5	0007	Chewing	4	0106	Gastric	4	0044	Delayed	9	0039	Dispersion	7
0060	Pastille	4	0008	Gargling	5	0107	Gastroenteral	5	0048	Unknown	10	0041	Mixing	7.5
0054	Gum	5	0017	Spraying	6	0108	Intestinal	6				0043	Unknown	10
0064	Pillules	6	0013	Instillation	7	0035	Rectal	7						
0062	Pellets	7	0015	Rinsing/washing	8	0036	Vaginal	8						
0053	Granules	7.2	0005	Application	9	0022	Cutaneous/transdermal	9						
0066	Powder	8	0006	Bathing	10	0021	Auricular	10						
0059	Lyophilisate	10	0012	Insertion	11	0029	Nasal	11						
0052	Film	11	0011	Injection	12	0030	Ocular	12						
0114	Herbal material (unprocessed)	12	0009	Infusion	13	0110	Oculonasal	13						
0070	Tea	13	0113	Implantation	14	0034	Pulmonary	14						
0085	Suspension	15	0010	Inhalation	15	0033	Parenteral	15						
0079	Dispersion	15.5	0111	Burning	16	0026	Intramammary	16						
0080	Emulsion	16	0112	Dialysis	17	0105	Endocervical	17						
0090	Drops	18	0004	Administration	18	0027	Intrauterine	18						
0082	Liquid	18.5	0020	Not specified	19	0028	Intravesical/urethral	19						
0084	Solvent	19				0109	Intraperitoneal	20						
0083	Solution	19.5				0024	Environmental	21						
0086	Syrup	20				0025	Extracorporeal	22						
0078	Concentrate	20.5				0037	Unknown/miscellaneous	23						
0094	Spray (unspecified)	22												
0081	Lacquer	23												
0077	Collodion	24												
0093	Shampoo	25												
0073	Gel	26												
0072	Foam	26.5												
0071	Cream	27												
0074	Ointment	29												
0076	Poultice	30												
0075	Paste	31												
0065	Plaster	33												
0061	Patch	33.5												
0056	Impregnated material	36												
0067	Stick	36.5												
0103	Cement	38												
0068	Suppository	40.5												
0063	Pessary	41												
0088	Insert	42												
0102	Pouch	42.5												
0055	Implant	43												
0089	Additive (unspecified)	44												
0092	Radiopharmaceutical	45												
0095	System	46												
0087	Medicinal gas	47												

**Table 2:** Standard Terms codification of some MPs containing risperidone.

Italian MA Number (AIC)	Description of Pharmaceutical Product	Basic Dose Form (BDF)	Administration Method (AME)	Intended Site (ISI)	Release Characteristics (RCA)	Transformation (TRN)	Number of DDDs * for Presentation Unit (NDXUP)
028752071	60; 3 mg film tablets	0069	0019	0031	0047	0042	0.6
028752095	100 mL; 1 mg/mL os solution	0083	0019	0031	0047	0042	0.2
037092069	60; 1 mg film tablets	0069	0019	0031	0047	0042	0.2
049100011	25 mg powder for solution + 2 mL solvent for IM injection	0085	0011	0033	0045	0039	1.85

* DDD = 5 mg for oral use, 2.7 mg for parenteral use.

**Table 3:** The string example of the new combined code for risperidone 3 mg film tablets, with 60 units per package.

ATC	BDF	AME	ISI	RCA	TRN	NDXUP
N05AX08	0069	0019	0031	0047	0042	0.6

**Table 4:** Penalty scores attributed to differences in number of DDDs for presentation unit (NDXUP) between lacking (lak) and potential substitute (sub) MPs.

Conditions	Penalty Score
NDXUPlak = NDXUPsub	0
0.5 × NDXUPlak = NDXUPsub	2
2 × NDXUPlak = NDXUPsub	4
0.5 × NDXUPlak < NDXUPsub < NDXUPlak	6
NDXUPlak < NDXUPsub < 2 × NDXUPlak	8
NDXUPsub < 0.5 × NDXUPlak	10
NDXUPsub > 2 × NDXUPlak	10

**Table 5:** Weight factors for each of the five STs considered.

Standard Term (ST)	Weight Factor (wf)
BDF	0.46
AME	0.18
ISI	0.20
RCA	0.09
TRN	0.07

**Table 6:** Examples of the official and proposed ATC codes of some illustrative FCs.

Italian MA Number (AIC)	API1	API2	Official ATC	New ATC
043496037	Rosuvastatin zinc 10 mg	Ezetimibe 10 mg	C10BA06	C10BA96
025253016	Desogestrel 0.15 mg	Ethinylestradiol 0.03 mg	G03AA09	G03AA09 *
029454028	Scopolamine butylbromide 10 mg	Paracetamol 800 mg	A03DB04	A03DB95
021462066	Amitriptyline hydrochloride 12.5 mg	Chlordiazepoxide 5 mg	N06CA01	N06CA95
021736020	Gentamycin sulphate 30 mg	Betamethasone valerate 30 mg	D07CC01	D07CC96

* The original code is unchanged, as it is unambiguous for this FC.

**Table 7:** DDDs of some illustrative FCs.

API1	DDD API1	API2	DDD API2	Sum of NDXUPs
Rosuvastatin zinc 10 mg	10 mg	Ezetimibe 10 mg	10 mg	2
Desogestrel 0.15 mg	1 tab	Ethinylestradiol 0.03 mg	25 mcg	0.15
Scopolamine butylbromide 10 mg	60 mg	Paracetamol 800 mg	3 g	0.43
Amitriptyline hydrochloride 12.5 mg	75 mg	Chlordiazepoxide 5 mg	30 mg	0.33
Gentamycin sulphate 30 mg	1 g	Betamethasone valerate 30 mg	2 g	45

**Table 8:** Output of the Excel macro based on this algorithm, searching for a substitute of the film tablet containing 2 mg of risperidone (Italian MA number 037599230).

Ranking Order	Italian MA Number (AIC)	MP Description	BDF	ISI	AME	RCA	TRA	NDXUP	DS Score
1	028752069	RISPERDAL 60TAB 2MG ORANGE	0069	0031	0019	0047	0042	0.4	**100**
2	037092222	RISPERIDONE TE 60FILM TAB 2MG	0069	0031	0019	0047	0042	0.4	**100**
3	040078293	RISPERIDONE AURO 60TAB 2MG	0069	0031	0019	0047	0042	0.4	**100**
4	040616082	RISPERIDONE MY 60FILM TAB 2MG	0069	0031	0019	0047	0042	0.4	**100**
5	028752057	RISPERDAL 60TAB 1MG WHITE	0069	0031	0019	0047	0042	0.2	**98.0**
6	037092069	RISPERIDONE TE 60FILM TAB 1MG	0069	0031	0019	0047	0042	0.2	**98.0**
7	037599065	RISPERIDONE SAN 60FILM TAB 1MG	0069	0031	0019	0047	0042	0.2	**98.0**
8	040078192	RISPERIDONE AURO 60TAB 1MG	0069	0031	0019	0047	0042	0.2	**98.0**
9	040616043	RISPERIDONE MY 60FILM TAB 1MG	0069	0031	0019	0047	0042	0.2	**98.0**
10	028752083	RISPERDAL 60TAB 4MG GREEN	0069	0031	0019	0047	0042	0.8	**96.0**
11	037092549	RISPERIDONE TE 60FILM TAB 4MG	0069	0031	0019	0047	0042	0.8	**96.0**
12	037599572	RISPERIDONE SAN 60FILM TAB 4MG	0069	0031	0019	0047	0042	0.8	**96.0**
13	040078495	RISPERIDONE AURO 60TAB 4MG	0069	0031	0019	0047	0042	0.8	**96.0**
14	040616207	RISPERIDONE MY 60FILM TAB 4MG	0069	0031	0019	0047	0042	0.8	**96.0**
15	028752071	RISPERDAL 60TAB 3MG YELLOW	0069	0031	0019	0047	0042	0.6	**92.0**
16	037092386	RISPERIDONE TE 60FILM TAB 3MG	0069	0031	0019	0047	0042	0.6	**92.0**
17	037599406	RISPERIDONE SAN 60FILM TAB 3MG	0069	0031	0019	0047	0042	0.6	**92.0**
18	040078394	RISPERIDONE AURO 60TAB 3MG	0069	0031	0019	0047	0042	0.6	**92.0**
19	040616120	RISPERIDONE MY 60FILM TAB 3MG	0069	0031	0019	0047	0042	0.6	**92.0**
20	037835030	RISPERIDONE SAND OS DROPS 100ML	0090	0031	0019	0047	0042	0.2	**84.4**
21	038188037	RISPERIDONE MY OS DROPS 100ML	0090	0031	0019	0047	0042	0.2	**84.4**
22	042441028	RISPERIDONE AURO DROPS 100ML	0090	0031	0019	0047	0042	0.2	**84.4**
23	028752095	RISPERDAL OS SOL 100ML 1MG/ML	0083	0031	0019	0047	0042	0.2	**83.2**
24	028752145	RISPERDAL OS SOL 30ML 1MG/ML	0083	0031	0019	0047	0042	0.2	**83.2**
25	049966017	OKEDI IM 1SYR 75MG RP	0085	0033	0011	0045	0042	13.9	**58.2**
26	049966029	OKEDI IM 1SYR 100MG RP	0085	0033	0011	0045	0042	18.5	**58.2**
27	028752172	RISPERDAL IM VL 25MG + 1SYR 2ML	0085	0033	0011	0045	0039	4.6	**55.7**
28	028752184	RISPERDAL IM VL 37.5MG + 1SYR 2ML	0085	0033	0011	0045	0039	6.9	**55.7**
29	028752196	RISPERDAL IM VL 50MG + 1SYR 2ML	0085	0033	0011	0045	0039	9.2	**55.7**
30	049100011	RISPERIDONE TE IM 1VL 25MG + 2ML	0085	0033	0011	0045	0039	1.8	**55.7**
31	049100047	RISPERIDONE TE IM VL 37.5MG + 2ML	0085	0033	0011	0045	0039	2.7	**55.7**
32	049100074	RISPERIDONE TE IM 1VL 50MG + 2ML	0085	0033	0011	0045	0039	3.7	**55.7**

**Table 9:** Feedback for external validation. Overall score from 1 (the lowest) to 5 (the highest).

Q&A	YES	NO	OTHER
Did you find the proposed solutions useful and practically exploitable?	100%	0%	0%
Was the file easy to consult?	43%	14%	43%
Have you been able to test the proposed solutions in clinical practice?	57%	43%	0%
Were the values returned by the algorithm useful?	71%	14%	14%
Were the results returned on treatment alternatives reliable?	86%	0%	14%
Overall, what is your assessment of the project (1–5 scale)?	MEDIUM SCORE = 4		

## Data Availability

The data used to create the algorithm described in this article will be made available by the authors on request.

## Supplementary Materials

The following supporting information can be downloaded at: https://www.mdpi.com/article/10.3390/healthcare13101139/s1, Table S1: Criteria for item selection for internal validation: the data of drug consumption in Italy for each ATC class are reported in comparison with European data (from AIFA report [27]); Table S2: List of official ATC codes, with the new codes introduced in cases of ambiguity.

## Abbreviations

The following abbreviations are used in this manuscript:

AIC	Italian acronym for the Italian marketing authorization
AIFA	Italian acronym for the Italian Medicines Agency
AME	Administration Method
API	Active pharmaceutical ingredient
ATC	Anatomical Therapeutical Chemical
BDF	Basic Dose Form
DDD	Defined Daily Dose
DS	Degree of substitutability
EDQM	European Directorate for the Quality of Medicines & HealthCare
EMA	European Medicines Agency
EU	European Union
FC	Fixed combination
FDA	Food and Drug Administration
ICH	International Council for Harmonisation of Technical Requirements for Pharmaceuticals for Human Use
ISI	Intended Site
MA	Marketing Authorization
MP	Medicinal product
NDXUP	Number of DDDs for presentation unit
NRD	Normalized relative distance
OTC	Over-the-counter
PCA	Principal Component Analysis
RCA	Release Characteristic
RP	Relative position
SSN	Italian acronym for the Italian Health Service
ST	Standard Term
ST-ID	Identification number of Standard Term
TRN	Transformation
USP	United States Pharmacopeia
WHO	World Health Organization
