# Supplementary material for: Medicine Shortages: An Algorithm for Evaluating the Substitution with Equivalent or Alternative Products
Source: Healthcare (Basel). 2025 May 14;13(10):1139. doi: 10.3390/healthcare13101139 (PMC12111303; doi:10.3390/healthcare13101139)
Supplement: Supplementary file 1 [file healthcare-13-01139-s001.zip › healthcare-3570257-supplementary.pdf]

Supplementary Materials

Table S1. Criteria for item selection for internal validation: the data of drug consumption in Italy for each ATC class are reported in comparison with European data (from AIFA report [27]).

ATC (1st level)	Drug consumption in Italy (%)	Drug consumption in Europe (%)	Number of MPs selected
C	26.9	19.0	161
A	17.4	17.3	104
N	15.1	17.1	91
R	14.2	22.3	85
M	6.9	5.7	41
B	5.4	4.7	32
H	4.0	2.7	24
G	2.9	2.4	17
S	2.6	2.7	16
D	2.3	3.0	14
J	1.7	1.7	10
L	0.4	0.7	2
V	0.2	0.5	1
P	0	0.2	0
Total items			598

Table S2. List of official ATC codes reporting the new codes introduced in case of ambiguity.

APIs Description	Official ATC	New ATC
SODIUM BENZOATE / AMYLOCAINE	A01AD11	A01AD99
LIDOCAINE / CETRIMIDE	A01AD11	A01AD98
PIOGLITAZONE HYDROCHLORIDE / METFORMIN HYDROCHLORIDE	A10BD05	A10BD95
PIOGLITAZONE / METFORMIN	A10BD05	A10BD96
SITAGLIPTIN HYDROCHLORIDE MONOHYDRATE / METFORMIN HYDROCHLORIDE	A10BD07	A10BD97
SITAGLIPTIN PHOSPHATE MONOHYDRATE / METFORMIN HYDROCHLORIDE	A10BD07	A10BD98
SITAGLIPTIN / METFORMIN HYDROCHLORIDE	A10BD07	A10BD99
FIBRINOGEN / APROTININ /HUMAN THROMBIN / CALCIUM CHLORIDE / FACTOR XIII	B02BC30	B02BC99
HUMAN FIBRINOGEN / HUMAN THROMBIN	B02BC30	B02BC98
APROTININ / HUMAN FIBRINOGEN / HUMAN THROMBIN / CALCIUM CHLORIDE	B02BC30	B02BC97
FACTOR II/FACTOR VII/FACTOR IX/FACTOR X OF COAGULATION/PROTEIN C/PROTEIN S	B02BD01	B02BD99
FACTOR II / FACTOR VII/ FACTOR IX / FACTOR X OF COAGULATION / PROTEIN C	B02BD01	B02BD98
SODIUM CHLORIDE / SODIUM ACETATE / SODIUM GLUCONATE / POTASSIUM CHLORIDE / MAGNESIUM CHLORIDE	B05BB01	B05BB84
SODIUM LACTATE / POTASSIUM CHLORIDE / SODIUM CHLORIDE	B05BB01	B05BB83
SODIUM CHLORIDE / POTASSIUM CHLORIDE / SODIUM BICARBONATE	B05BB01	B05BB82
SODIUM CHLORIDE / POTASSIUM CHLORIDE / CALCIUM CHLORIDE / MAGNESIUM CHLORIDE / SODIUM ACETATE / SODIUM CITRATE	B05BB01	B05BB81
SODIUM CHLORIDE / POTASSIUM CHLORIDE / CALCIUM CHLORIDE	B05BB01	B05BB80
SODIUM CHLORIDE / POTASSIUM CHLORIDE / CALCIUM CHLORIDE /SODIUM ACETATE	B05BB01	B05BB79
LACTIC ACID / SODIUM HYDROXIDE / SODIUM CHLORIDE / POTASSIUM CHLORIDE	B05BB01	B05BB78
SODIUM CHLORIDE / POTASSIUM CHLORIDE / CALCIUM CHLORIDE / MAGNESIUM CHLORIDE / SODIUM ACETATE	B05BB01	B05BB77

SODIUM CHLORIDE / POTASSIUM CHLORIDE / AMMONIUM CHLORIDE	B05BB01	B05BB76
POTASSIUM CHLORIDE / SODIUM CHLORIDE	B05BB01	B05BB75
SODIUM CHLORIDE/POTASSIUM CHLORIDE/MAGNESIUM CHLORIDE HEXAHYDRATE/ CALCIUM CHLORIDE DIHYDRATE / SODIUM ACETATE TRIHYDRATE / MALIC ACID	B05BB01	B05BB74
SODIUM ACETATE TRIHYDRATE / SODIUM CHLORIDE / POTASSIUM CHLORIDE / MAGNESIUM CHLORIDE HEXAHYDRATE	B05BB01	B05BB73
SODIUM CHLORIDE / POTASSIUM CHLORIDE / MAGNESIUM CHLORIDE HEXAHYDRATE/ SODIUM ACETATE TRIHYDRATE / SODIUM GLUCONATE	B05BB01	B05BB72
GLUCOSE (DEXTROSE) ANHYDROUS / SODIUM ACETATE / SODIUM CHLORIDE / POTASSIUM CHLORIDE / MAGNESIUM CHLORIDE	B05BB02	B05BB95
GLUCOSE (DEXTROSE) ANHYDROUS / SODIUM ACETATE / SODIUM CHLORIDE / POTASSIUM CHLORIDE / MAGNESIUM CHLORIDE / CALCIUM CHLORIDE / ACETIC ACID	B05BB02	B05BB94
GLUCOSE (DEXTROSE) ANHYDROUS / SODIUM CHLORIDE	B05BB02	B05BB93
POTASSIUM CHLORIDE / GLUCOSE (DEXTROSE) MONOHYDRATE	B05BB02	B05BB92
SODIUM ACETATE / POTASSIUM CHLORIDE / MAGNESIUM CHLORIDE / DIPOTASSIUM PHOSPHATE / GLUCOSE (DEXTROSE) MONOHYDRATE	B05BB02	B05BB91
SODIUM CHLORIDE / POTASSIUM ACETATE / MAGNESIUM ACETATE / GLUCOSE (DEXTROSE) MONOHYDRATE	B05BB02	B05BB90
SODIUM CHLORIDE / POTASSIUM ACETATE / DIPOTASSIUM PHOSPHATE / MAGNESIUM SULFATE HEPTAHYDRATE / CALCIUM GLUCONATE MONOHYDRATE /GLUCOSE (DEXTROSE) MONOHYDRATE	B05BB02	B05BB89
SODIUM CHLORIDE / POTASSIUM CHLORIDE / CALCIUM CHLORIDE / MAGNESIUM CHLORIDE / SODIUM ACETATE / SODIUM CITRATE / GLUCOSE (DEXTROSE) MONOHYDRATE	B05BB02	B05BB88
SODIUM CHLORIDE / POTASSIUM CHLORIDE / DIPOTASSIUM PHOSPHATE / SODIUM ACETATE / GLUCOSE (DEXTROSE) MONOHYDRATE	B05BB02	B05BB87
SODIUM CHLORIDE / POTASSIUM CHLORIDE / MAGNESIUM CHLORIDE / POTASSIUM ACETATE / SODIUM ACETATE / SODIUM GLUCONATE / GLUCOSE (DEXTROSE) MONOHYDRATE	B05BB02	B05BB86
SODIUM CHLORIDE / POTASSIUM CHLORIDE / MAGNESIUM CHLORIDE / SODIUM ACETATE / SODIUM GLUCONATE / GLUCOSE (DEXTROSE) MONOHYDRATE	B05BB02	B05BB85
AMILORIDE HYDROCHLORIDE / HYDROCHLOROTHIAZIDE	C03EA01	C03EA95
SPIRONOLACTONE / HYDROCHLOROTHIAZIDE	C03EA01	C03EA96
FUROSEMIDE / SPIRONOLACTONE	C03EB01	C03EB95
FUROSEMIDE / TRIAMTERENE	C03EB01	C03EB96
HYDROCORTISONE / ESCULIN / AMILINE / BENZOCAINE / BENZALKONIUM CHLORIDE	C05AA01	C05AA99
HYDROCORTISONE / ESCULIN / BENZOCAINE / BENZALKONIUM CHLORIDE	C05AA01	C05AA98
HYDROCORTISONE / BENZOCAINE / SODIUM HEPARIN	C05AA01	C05AA97
LIDOCAINE HYDROCHLORIDE / HYDROCORTISONE ACETATE	C05AA01	C05AA96
ATENOLOL / CHLORTALIDONE	C07CB03	C07CB95
ATENOLOL / INDAPAMIDE	C07CB03	C07CB96
PERINDOPRIL ARGININE/INDAPAMIDE	C09BA04	C09BA95
PERINDOPRIL ERBUMINE (TERT-BUTYLAMINE) / INDAPAMIDE	C09BA04	C09BA96
PERINDOPRIL TOSILATE / INDAPAMIDE	C09BA04	C09BA97
RAMIPRIL / HYDROCHLOROTHIAZIDE	C09BA05	C09BA98
RAMIPRIL / PYRETHANIDE	C09BA05	C09BA99
PERINDOPRIL ARGININE / AMLODIPINE BESYLATE	C09BB04	C09BB95
PERINDOPRIL ERBUMINE (TERT-BUTYLAMINE) / AMLODIPINE BESYLATE	C09BB04	C09BB96
PERINDOPRIL TOSILATE / AMLODIPINE BESYLATE	C09BB04	C09BB97
PERINDOPRIL ARGININE / INDAPAMIDE / AMLODIPINE BESYLATE	C09BX01	C09BX95

PERINDOPRIL ERBUMINE / INDAPAMIDE / AMLODIPINE BESYLATE	C09BX01	C09BX96
IRBESARTAN HYDROCHLORIDE / HYDROCHLOROTHIAZIDE	C09DA04	C09DA95
IRBESARTAN / HYDROCHLOROTHIAZIDE	C09DA04	C09DA96
ROSUVASTATIN CALCIUM SALT / EZETIMIBE	C10BA06	C10BA95
ROSUVASTATIN ZINC / EZETIMIBE	C10BA06	C10BA96
ROSUVASTATIN CALCIUM SALT / ACETYLSALICYLIC ACID	C10BX05	C10BX95
ROSUVASTATIN / ACETYLSALICYLIC ACID	C10BX05	C10BX96
CLOTRIMAZOLE / DEXAMETHASONE	D01AC20	D01AC99
ECONAZOLE NITRATE / TRIAMCINOLONE ACETONIDE	D01AC20	D01AC98
ISOCONAZOLE / DIFLUCORTOLONE	D01AC20	D01AC97
MICONAZOLE / FLUPREDNIDENE ACETATE	D01AC20	D01AC96
SILVER SULFADIAZINE / SODIUM HYALURONATE	D03AX	D0399
CATALASE / GENTAMICIN	D03AX	D0398
TRIAMCINOLONE / CHLORTETRACYCLINE	D07CB01	D07CB95
TRIAMCINOLONE / NEOMYCIN	D07CB01	D07CB96
FUSIDIC ACID / BETAMETHASONE VALERATE	D07CC01	D07CC95
GENTAMICIN SULFATE / BETAMETHASONE VALERATE	D07CC01	D07CC96
FLUOCINOLONE ACETONIDE / ERYTHROMYCIN STEARATE	D07CC02	D07CC97
FLUOCINOLONE ACETONIDE / NEOMYCIN	D07CC02	D07CC98
BETAMETHASONE / SALICYLIC ACID	D07XC01	D07XC99
AMMONIUM BITUMEN SULFONATE / SALICYLIC ACID / BETAMETHASONE VALERATE ACETATE	D07XC01	D07XC98
EOSIN / CHLOROXYLENOL / PROPYLENE GLYCOL	D08AX	D0899
SALICYLIC ACID / SODIUM IODIDE	D08AX	D0898
BENZOYL PEROXIDE / CLINDAMYCIN PHOSPHATE	D10AF51	D10AF99
CLINDAMYCIN PHOSPHATE / TRETINOIN	D10AF51	D10AF98
DROSPIRENONE / ETHINYL ESTRADIOL	G03AA12	G03AA95
DROSPIRENONE / ETHINYL ESTRADIOL BETADEX CLATHRATE	G03AA12	G03AA96
AMOXICILLIN SODIUM / POTASSIUM CLAVULANATE	J01CR02	J01CR95
AMOXICILLIN TRIHYDRATE / POTASSIUM CLAVULANATE	J01CR02	J01CR96
PIPERACILLIN SODIUM / TAZOBACTAM SODIUM	J01CR05	J01CR97
PIPERACILLIN / TAZOBACTAM	J01CR05	J01CR98
EMTRICITABINE / TENOFOVIR DISOPROXIL PHOSPHATE	J05AR03	J05AR94
EMTRICITABINE / TENOFOVIR DISOPROXIL FUMARATE	J05AR03	J05AR93
EMTRICITABINE / TENOFOVIR DISOPROXIL MALEATE	J05AR03	J05AR92
EMTRICITABINE / TENOFOVIR DISOPROXIL SUCCINATE	J05AR03	J05AR91
EFAVIRENZ / EMTRICITABINE / TENOFOVIR DISOPROXIL	J05AR06	J05AR90
EFAVIRENZ / EMTRICITABINE / TENOFOVIR DISOPROXIL PHOSPHATE	J05AR06	J05AR89
EFAVIRENZ / EMTRICITABINE / TENOFOVIR DISOPROXIL MALEATE	J05AR06	J05AR88
EFAVIRENZ / EMTRICITABINE / TENOFOVIR DISOPROXIL SUCCINATE	J05AR06	J05AR87
SODIUM ALENDRONATE MONOHYDRATE / CHOLECALCIFEROL	M05BB03	M05BB95
SODIUM ALENDRONATE TRIHYDRATE / CHOLECALCIFEROL	M05BB03	M05BB96
NEOMYCIN / FLUOCINOLONE ACETONIDE / LIDOCAINE	N01BB52	N01BB99
LIDOCAINE / TETRACAINE	N01BB52	N01BB98
LIDOCAINE HYDROCHLORIDE / ADRENALINE BITARTRATE	N01BB52	N01BB97
LIDOCAINE / CETRIMONIUM BROMIDE	N01BB52	N01BB96
PARACETAMOL / CODEINE PHOSPHATE	N02AJ06	N02AJ95

PARACETAMOL / CODEINE PHOSPHATE HEMIHYDRATE	N02AJ06	N02AJ96
TRAMADOL HYDROCHLORIDE / DEXKETOPROFEN	N02AJ14	N02AJ97
TRAMADOL HYDROCHLORIDE / DEXKETOPROFEN TROMETHAMOL	N02AJ14	N02AJ98
ACETYLSALICYLIC ACID / SODIUM BICARBONATE / ANHYDROUS CITRIC ACID	N02BA51	N02BA99
ACETYLSALICYLIC ACID / ASCORBIC ACID	N02BA51	N02BA98
PARACETAMOL / ACETYLSALICYLIC ACID / CAFFEINE	N02BE51	N02BE80
PARACETAMOL / PROPYPHENAZONE / CAFFEINE	N02BE51	N02BE81
PARACETAMOL / ACETYLSALICYLIC ACID / ASCORBIC ACID	N02BE51	N02BE82
SOBREROLO / PARACETAMOL	N02BE51	N02BE83
PARACETAMOL / ASCORBIC ACID	N02BE51	N02BE84
PARACETAMOL / ACETYLSALICYLIC ACID	N02BE51	N02BE85
PARACETAMOL / ASCORBIC ACID / PHENYLEPHRINE HYDROCHLORIDE	N02BE51	N02BE86
PARACETAMOL / CHLORPHENAMINE MALEATE / SODIUM ASCORBATE	N02BE51	N02BE87
PARACETAMOL / CHLORPHENAMINE MALEATE	N02BE51	N02BE88
PARACETAMOL / PSEUDOEPHEDRINE HYDROCHLORIDE / DIPHENHYDRAMINE HYDROCHLORIDE	N02BE51	N02BE89
PARACETAMOL / GUAIFENESIN / PHENYLEPHRINE HYDROCHLORIDE	N02BE51	N02BE90
PARACETAMOL / PHENYLEPHRINE HYDROCHLORIDE	N02BE51	N02BE91
PARACETAMOL / DEXTROMETHORPHAN / CHLORPHENAMINE	N02BE51	N02BE92
PARACETAMOL / CAFFEINE	N02BE51	N02BE93
PARACETAMOL / IBUPROFEN	N02BE51	N02BE94
PARACETAMOL / THIOCOLCHICOSIDE	N02BE51	N02BE95
PARACETAMOL / IBUPROFEN SODIUM DIHYDRATE	N02BE51	N02BE96
ERGOTAMINE / CAFFEINE	N02CA52	N02CA99
ERGOTAMINE TARTRATE / CAFFEINE / AMINOPHENAZONE	N02CA52	N02CA98
BUXAMINE / PHENOBARBITAL / PHENYTOIN	N03AG	N0399
BUXAMINE / DIAZEPAM	N03AG	N0398
LEVODOPA / CARBIDOPA	N04BA02	N04BA94
LEVODOPA / BENSERAZIDE HYDROCHLORIDE	N04BA02	N04BA95
AMITRIPTYLINE HYDROCHLORIDE / CHLORDIAZEPOXIDE	N06CA01	N06CA95
AMITRIPTYLINE / PERPHENAZINE	N06CA01	N06CA96
EPHEDRINE HYDROCHLORIDE / NAFAZOLINE	R01AB05	R01AB99
SILVER VITELLINATE / EPHEDRINE HYDROCHLORIDE	R01AB05	R01AB98
MENTHOL / CAMPHOR	R01AX10	R01AX99
NEOMYCIN / EUCALYPTOL /PINE ESSENCE / CAMPHOR/ MENTHOL/CHLOROBUTANOL	R01AX10	R01AX98
PSEUDOEPHEDRINE HYDROCHLORIDE / TRIPROLIDINE HYDROCHLORIDE	R01BA52	R01BA99
PARACETAMOL / PSEUDOEPHEDRINE HYDROCHLORIDE / TRIPROLIDINE HYDROCHLORIDE	R01BA52	R01BA98
CETIRIZINE DICHLORIDE / PSEUDOEPHEDRINE HYDROCHLORIDE	R01BA52	R01BA97
PARACETAMOL / PSEUDOEPHEDRINE HYDROCHLORIDE	R01BA52	R01BA96
ACETYLSALICYLIC ACID / PSEUDOEPHEDRINE HYDROCHLORIDE	R01BA52	R01BA95
DESLORATADINE / PSEUDOEPHEDRINE SULFATE	R01BA52	R01BA94
BENZYL ALCOHOL / SODIUM BENZOATE	R02AA20	R02AA99
DICHLOROPHENYLCARBINOL / AMILMETHACRESOL/ SODIUM ASCORBATE/ ASCORBIC ACID	R02AA20	R02AA98
DICHLOROPHENYLCARBINOL / SODIUM BENZOATE	R02AA20	R02AA97
DICHLOROPHENYLCARBINOL / ASCORBIC ACID	R02AA20	R02AA96

BENZIDAMINE HYDROCHLORIDE / CETYLPYRIDINIUM CHLORIDE	R02AA20	R02AA95
AMILMETHACRESOL / 2,4-DICHLOROBENZYL ALCOHOL	R02AA20	R02AA94
BECLOMETASONE DIPROPIONATE /SALBUTAMOL SULFATE	R03AK13	R03AK95
BECLOMETASONE / SALBUTAMOL	R03AK13	R03AK96
TRIPROLIDINE HYDROCHLORIDE / PSEUDOEPHEDRINE HYDROCHLORIDE / DEXTROMETHORPHAN BROMIDE	R05DA20	R05DA99
DEXTROMETHORPHAN BROMIDE / DOXYLAMINE SUCCINATE / PARACETAMOL	R05DA20	R05DA98
DEXTROMETHORPHAN BROMIDE / DICHLOROPHENYLCARBINOL	R05DA20	R05DA97
DIHYDROCODEINE / BENZOIC ACID	R05DA20	R05DA96
PENTETRAZOL / DIHYDROCODEINE RHODANATE	R05DA20	R05DA95
DEXTROMETHORPHAN BROMIDE / GUAIFENESIN	R05FA02	R05FA95
SULFOGUAICOL / DEXTROMETHORPHAN BROMIDE	R05FA02	R05FA96
TETRACYCLINE / SULFAMETHIAZOLE	S01AA30	S01AA99
CHLORAMPHENICOL / COLISTIMETHATE SODIUM / TETRACYCLINE HYDROCHLORIDE	S01AA30	S01AA98
TOBRAMYCIN / DESAMETHASONE	S01CA01	S01CA95
TOBRAMYCIN / DESAMETHASONE SODIUM PHOSPHATE	S01CA01	S01CA95
DESAMETHASONE PHOSPHATE DISODIUM / NETILMICIN SULFATE	S01CA01	S01CA95
DESAMETHASONE SODIUM PHOSPHATE / LEVOFLOXACIN HEMIHYDRATE	S01CA01	S01CA96
DESAMETHASONE / CHLORAMPHENICOL	S01CA01	S01CA97
BETAMETHASONE SODIUM PHOSPHATE / CHLORAMPHENICOL	S01CA05	S01CA95
BETAMETHASONE / CHLORAMPHENICOL	S01CA05	S01CA96
BETAMETHASONE / CHLORAMPHENICOL / TETRACYCLINE /COLISTIMETHATE SODIUM	S01CA05	S01CA97
BETAMETHASONE / SULFACETAMIDE	S01CA05	S01CA98
BRINZOLAMIDE / TIMOLOL MALEATE	S01EC54	S01EC99
BRINZOLAMIDE / BRIMONIDINE TARTRATE	S01EC54	S01EC98
TIMOLOL MALEATE / PILOCARPINE HYDROCHLORIDE	S01ED51	S01ED99
DORZOLAMIDE HYDROCHLORIDE / TIMOLOL MALEATE	S01ED51	S01ED98
LATANOPROST / TIMOLOL MALEATE	S01ED51	S01ED97
TIMOLOL MALEATE / BRIMONIDINE TARTRATE	S01ED51	S01ED96
TIMOLOL MALEATE / TRAVOPROST	S01ED51	S01ED95
TIMOLOL MALEATE / BIMATOPROST	S01ED51	S01ED94
TAFLUPROST / TIMOLOL MALEATE	S01ED51	S01ED93
TROPICAMIDE / PHENYLEPHRINE HYDROCHLORIDE	S01FA56	S01FA99
TROPICAMIDE / PHENYLEPHRINE HYDROCHLORIDE / LIDOCAINE HYDROCHLORIDE	S01FA56	S01FA98
NAPHAZOLINE / ZINC PHENOLSULFONATE	S01GA51	S01GA99
NAPHAZOLINE / TONZILAMINE	S01GA51	S01GA98
METHYLHYDROXYPROPYLCELLULOSE / BENZYALKONIUM CHLORIDE	S01XA20	S01XA97
LIQUID PARAFFIN / SOFT PARAFFIN	S01XA20	S01XA98
BENZALKONIUM CHLORIDE / POLYACRYLIC ACID	S01XA20	S01XA95
BENZALKONIUM CHLORIDE / METHYLCELLULOSE	S01XA20	S01XA96
CIPROFLOXACIN HYDROCHLORIDE MONOHYDRATE / HYDROCORTISONE	S02CA03	S02CA95
HYDROCORTISONE / POLYMYXIN B / NEOMYCIN / LIDOCAINE	S02CA03	S02CA96
